# Magnetic resonance thermometry in the target volume versus intraluminal probe thermometry for hyperthermia treatment monitoring

**DOI:** 10.1016/j.phro.2025.100812

**Published:** 2025-07-24

**Authors:** Carolina Carrapiço-Seabra, Spyridon N. Karkavitsas, Anton Rink, Nahid Montazeri, Henrike Westerveld, Martine Franckena, Margarethus M. Paulides, Gerard C. van Rhoon, Sergio Curto

**Affiliations:** a Department of Radiotherapy, Erasmus MC Cancer Institute, University Medical Center Rotterdam, Rotterdam, the Netherlands; b Department of Medicine III, LMU University Hospital, LMU Munich, Munich, Germany; c Department of Hematology, Erasmus MC Cancer Institute, University Medical Center Rotterdam, Rotterdam, the Netherlands; d Care+Cure lab of the Electromagnetics group (EM4C+C), Department of Electrical Engineering, Eindhoven University of Technology, Eindhoven, the Netherlands; e Department of Radiation Science and Technology, Faculty of Applied Sciences, Delft University of Technology, Delft, the Netherlands

**Keywords:** Hyperthermia, Locally advanced cervical cancer, Magnetic resonance-guided hyperthermia, Probe thermometry, Magnetic resonance thermometry, Target volume temperature, Repeated measures correlation

## Abstract

**Background and purpose:**

Hyperthermia, the elevation of target temperature to 39–44 °C, is monitored using temperature probes. However, these provide limited spatial information, sampling only a few discrete locations. Magnetic resonance (MR) thermometry currently offers an option for three-dimensional (3D) temperature monitoring during hyperthermia. This study compares and correlates temperatures measured by intraluminal probes with MR-based temperatures in (1) the anatomical region containing the intraluminal probes and (2) the hyperthermia target volume (HTV), located at a distance from the probes and representing the primary region of clinical interest.

**Methods:**

Thirteen locally advanced cervical cancer (LACC) patients treated with radiotherapy and hyperthermia were included. Hyperthermia was monitored using intraluminal probes and MR thermometry. MR-based temperatures were compared to intraluminal probe temperatures. Repeated measures correlation was applied to correlate probe and MR-based temperatures in the HTV across all data and on a patient-specific basis.

**Results:**

MR-based temperatures at probe locations showed good agreement with probe measurements (median absolute error ≤ 0.7 °C). In the HTV, MR-based temperatures deviated by a median absolute error of 0.5 °C from probe temperatures. Repeated measures correlations (r_rm_) between MR and probe-based HTV temperatures ranged from 0.74 to 0.79 across all data and 0.64–0.96 on a patient-specific basis.

**Conclusions:**

MR thermometry demonstrated promising performance for retrospective evaluation of temperature distributions in the HTV. While its current reliability for real-time treatment guidance remains limited, our results support further development towards broader clinical implementation in hyperthermia.

## Highlights

• Magnetic resonance thermometry at probe sites showed median absolute errors within 0.7 °C.
• Magnetic resonance temperatures in the target volume were comparable to probe measurements.
• Correlations between magnetic resonance and probe temperatures ranged from 0.74–0.79 overall and 0.64–0.96 per patient.
• Magnetic resonance thermometry was feasible for retrospective target temperature evaluation.
• Further improvements are needed for real-time treatment guidance.

## 1 Introduction

Hyperthermia is a cancer treatment that enhances the efficacy of radiotherapy and chemotherapy [[1], [2], [3], [4]]. It aims to increase the target temperature to 39–44 °C for 60–90 min [[5], [6], [7]]. Multiple studies have reported correlations between treatment outcomes and thermal parameters achieved during treatment [8,9]. Franckena et al. [10] showed that higher intraluminally measured thermal parameters correlated with tumour control and disease-specific survival in a retrospective analysis of 420 patients with locally advanced cervical cancer (LACC). A more recent study confirmed the predictive value of thermal dose for local control in an independent cohort of LACC patients treated with hyperthermia and state-of-the-art radiotherapy [11]. Several studies [8,9] have stressed the importance of temperature measurements during hyperthermia, both to guide treatments and to ensure adequate heat delivery [12,13].

In clinical practice, temperatures are monitored using thermal probes placed within the patient’s body [14,15]. While effective, thermal probes have limited spatial resolution, measuring temperatures only at discrete locations along the implanted catheter [16,17]. To address this, alternative non-invasive, three-dimensional (3D) thermometry approaches have been developed over the last decades. Among them, magnetic resonance-guided hyperthermia (MRgHT) is currently the most clinically used approach, integrating MR thermometry with conventional hyperthermia [18,19]. This allows acquisition of 3D temperature maps across the entire treatment region, including not only the tumour, but also healthy tissues. Hence, MR temperature monitoring potentially encourages dynamic treatment delivery, as extended thermal dosimetry and optimisation may be performed.

The feasibility of MR thermometry using the proton resonance frequency shift (PRFS) method, has been demonstrated both in phantoms and patients [[20], [21], [22], [23], [24], [25], [26]]. Craciunescu et al. [23] reported temperature differences below 1 °C between probe and MR-based measurements in extremity sarcomas. Similarly, Gellermann et al. [22] observed a correlation of 0.96 between MR and probe thermometry readings in patients with sarcoma of the lower extremities and pelvis. A recent study by Unsoeld et al. [26] reported a correlation between MR temperatures and pathological response in patients with sarcomas of the lower extremities. Some groups have also evaluated the performance of MR thermometry in areas sensitive to air motion, such as pelvic tumours [[27], [28], [29]]. Of particular interest is the study of VilasBoas-Ribeiro et al. [29], which evaluated the accuracy of MR temperatures in intraluminal probe regions in patients with LACC. Their findings showed that, for a selected group of patients, MR thermometry yielded values within the recommended minimal requirements (accuracy ≤ 1 °C, bias ≤ |0.5 °C|, precision ≤ 0.5 °C) [30]. However, that study focused exclusively on intraluminal probes and did not evaluate the target volume, thus limiting the applicability of its findings to other regions.

In this study, we evaluated MR-based temperature in two relevant locations: (1) at intraluminal probe positions, which served as reference points for validation and (2) in the HTV, where direct temperature measurements are unavailable, yet where temperature assessment is of primary clinical importance. While the comparison at the probe locations was included to confirm baseline performance, the aim of this study was to assess whether MR thermometry can retrospectively provide consistent and representative temperature estimates within the HTV over the course of the entire treatment session. To address this, we conducted a correlation-based analysis between MR-derived and probe temperatures over the entire treatment duration. This approach allowed us to examine how well MR thermometry retrospectively reflects clinically relevant thermal patterns that, in practice, are used to guide treatment adjustments in real time. Furthermore, to assess inter-patient variability, a patient-specific correlation analysis was performed. To the best of our knowledge, this study represents the first comprehensive evaluation of 3D MR thermometry in both surrogate and target regions in patients with LACC.

## 2 Materials and methods

### 2.1 Patient cohort

All patients diagnosed with LACC who received radiotherapy combined with (at least one) MRgHT treatment with curative intent at the Erasmus Medical Center (MC) Cancer Institute between February 2017 and 2019 were included (Table S1, Supplementary Materials). Patients received external beam radiotherapy (EBRT) combined with weekly hyperthermia, followed by a brachytherapy boost. EBRT consisted of 23–25 daily fractions of 1.8–2.0 Gray (Gy) per fraction, with an additional boost to 60 Gy in EQD2_αβ10_ for patients with pathological lymph nodes_._ MR image-guided adaptive brachytherapy consisted of 3–4 fractions of 7 Gy delivered over 2–3 applications. The study was approved by the Erasmus MC ethics committee (MEC 2015-108).

### 2.2 Hyperthermia treatment

Hyperthermia was administered once weekly following EBRT, for five sessions over the five-week EBRT period. Treatments were delivered using the BSD-2000-3D MR-compatible applicator (Pyrexar Medical Corp., Salt Lake City, UT, USA), operating at 100 MHz and positioned within the bore of a 1.5T GE Signa Excite scanner (General Electric Healthcare, Waukesha, WI, USA), thereby allowing MR imaging during treatment. Although the MR-compatible applicator was preferred to facilitate MR thermometry, its use was occasionally restricted due to technical limitations or, more commonly, patient discomfort. Consequently, of the 64 hyperthermia treatments delivered to the 13 included patients, only 37 were performed using the MR-compatible BSD-2000-3D applicator, while the remaining were conducted using the non-MR version of the device. On average, each patient received 4.9 ± 0.3 hyperthermia treatments, of which 2.8 ± 1.5 were MRgHT (Supplementary Materials, Fig. S1). Given the focus on MR thermometry, only MRgHT treatments were included in the analysis.

Temperature monitoring was performed using intraluminal thermometry with single sensor thermistor probes (Pyrexar Medical Corporation, Salt Lake City, Utah, USA). These probes were placed in closed-tip catheters in the bladder, rectum and vagina for all patients. Thermal mapping was performed with 1 cm step size and a minimum and maximum mapping length of 3 cm and 14 cm, respectively. Temperatures were obtained by calculating the average temperature along the entire length of each probe track. Rectal and vaginal probe temperatures were available for all treatments, while bladder probe temperatures were unavailable for three treatments in two patients due to discomfort to bladder catheter placement. The planned treatment duration was 90 min. The initial 30 min corresponded to the heating-up phase, during which temperatures were increased to the therapeutic range. The subsequent 60 min corresponded to the steady-state phase, where therapeutic temperatures were maintained. Patients were instructed to report any discomfort suggestive of undesirable hotspots. When complaints occurred, treatment settings, as phase and amplitude were adjusted to alleviate the complaints [31].

### 2.3 Clinical MR protocol

**Fig. 1:**
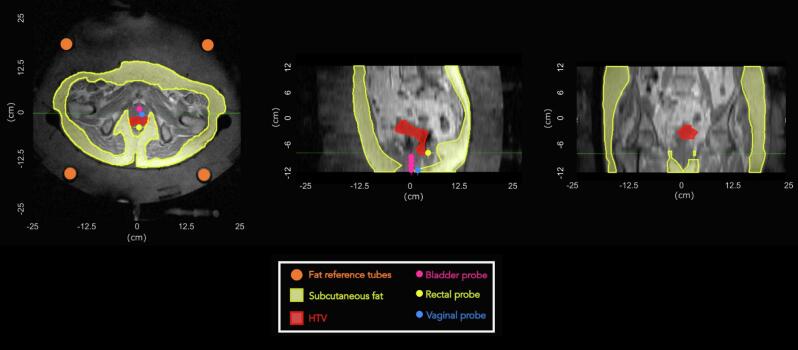
Axial (left) and sagittal (middle) and coronal (right) views of the T1-weighted MR image. Fat reference tubes and subcutaneous fat are delineated in orange and yellow, respectively. The delineation of the HTV is shown in red, while the locations of the thermal probes are shown in magenta, yellow and blue, for bladder, rectal and vaginal probes, respectively. (HTV: hyperthermia target volume). (For interpretation of the references to colour in this figure legend, the reader is referred to the web version of this article.)

The MR protocol consisted of gradient recalled echo (GRE) sequences with two echo times (dual echo, DE) as provided by the manufacturer (Dr. Sennewald Medizintechnik GmbH, Munchen, Germany). A high-resolution T1-weighted scan was acquired at the beginning of each treatment for anatomical and position verification and for segmentation of fat tube references and subcutaneous body fat for postprocessing correction (Fig. 1). Parameters of this scan were: TR = 120 ms; TE1 = 4.8 ms; TE2 = 9.6 ms; flip angle = 70°; field of view (FOV) = 50 × 50 cm^2^; acquisition matrix = 256 × 256; reconstruction matrix = 256 × 256; slice thickness = 1 cm; number of slices = 25; acquisition time (mm:ss) = 02:16.

Additional DE-GRE scans were performed twice before powering on and, then, approximately every 10 min, during treatment. Scan parameters were the following: TR = 620 ms; TE1 = 4.8 ms; TE2 = 19.1 ms; flip angle = 40°; FOV = 50 × 50 cm^2^; acquisition matrix = 128 × 128; reconstruction matrix = 256 × 256; slice thickness = 1 cm; number of slices = 25; acquisition time (mm:ss) = 01:23. These DE-GRE scans were used for 3D temperature calculations.

### 2.4 Delineations for temperature calculation

The baseline MR image from each session was used to locate catheters containing the thermal probes (details in the Supplementary Materials). The delineated probe regions of interest (ROIs) (Fig. 1) were subsequently utilised to calculate MR-based temperatures at the probe locations, hereafter referred to as MR temperatures at probe locations.

The hyperthermia target volume (HTV) was defined using the planning computed tomography (CT) images (Supplementary Materials, Fig. S2). The HTV delineation (Fig. 1) was then used to calculate all MR-based temperatures in the target, hereafter referred as MR temperatures in the HTV.

Moreover, subcutaneous fat volume was delineated in the same MR image for each treatment session of each patient (Fig. 1), being subsequently used for MR thermometry processing.

### 2.5 MR thermometry processing

To obtain temperatures from the MR images, the PRFS method was applied [32,33] (details provided in Supplementary Materials). Average MR temperatures were calculated for each ROI (probe locations and HTV). All average MR-based temperatures were compared against intraluminal probe temperatures for the corresponding time points. For these calculations, a threshold of 0–6 °C was applied (Supplementary Materials, Fig. S3), leading to the exclusion of pixels from the probe and HTV ROIs. Two variables were defined: the percentage of pixels used to calculate the MR temperature at the probe locations and in the HTV.

### 2.6 Statistical analysis

Statistical analyses were performed using R statistical software (v4.1.1) [34] and an alpha level of 0.05 was used for all analyses.

Repeated measures correlation (rmcorr package [35]) assessed the relationship between MR-based temperatures in the HTV and probe temperatures across patients and treatments, accounting, thus, for inter-patient variability and the interdependence of repeated measurements. Additionally, each patient's repeated measures correlation was computed individually for intra-treatment analysis. For patients who underwent only one treatment, a simple Pearson correlation was applied. For patients with multiple treatments, repeated measures correlation was applied [35]. For simple correlations *r* was analysed, while for repeated measures *r_rm_* was analysed, both with corresponding 95 % confidence interval and p-value.

A linear mixed-effects model (lme package [36]) was applied to test associations between MR temperatures in the HTV and: (i) HTV volume, (ii) subcutaneous fat volume and (iii) percentage of pixels used to calculate the MR temperature in the HTV.

## 3 Results

Hyperthermia temperature-related parameters for both probe and MR thermometry are summarised in Table 1. Additional details are given in Table S2 (Supplementary Materials).

**Table 1:** Temperature-related parameters for both probe and MR thermometry. All data are presented as frequency count, mean ± standard deviation or median (interquartile range, IQR) as appropriate. Parameters were obtained for the entire treatment duration, except when steady-state is mentioned. (MR: magnetic resonance; HTV: hyperthermia target volume).

Parameter	Value (mean ± std or median (IQR))
Total number of treatments	37
Duration of each treatment session (minutes)	89.0 ± 1.5
Net* heating power (W)	602.5 ± 101.0
Total number of MR thermometry scans per treatment	321 9 ± 2

Mapping length per probe (cm)	
Bladder	9.9 ± 2.1
Rectum	7.0 ± 2.0
Vagina	8.2 ± 2.2
All probes	8.3 ± 2.5
Number of probe mappings	15.2 ± 3.0
With a time interval (minutes)	5.0 (5.0–5.3)

Intraluminal probe temperature (°C)	
Bladder	40.2 (38.9–40.9)
Rectum	39.8 (38.5–40.5)
Vagina	39.9 (38.7–40.5)
All probes	40.0 (38.6–40.6)

MR temperature at probe location (°C)	
Bladder	40.1 (38.8–40.9)
Rectum	39.7 (38.6–40.7)
Vagina	40.1 (38.8–40.8)
All probes	40.0 (38.7–40.8)
MR temperature in the HTV (°C)	40.4 (39.5–40.8)

Probe ROI volume (cm^3^)	
Bladder	14.7 ± 5.6
Rectum	13.8 ± 2.7
Vagina	16.7 ± 4.2
All probes	15.1 ± 4.5

Percentage of pixels used to calculate MR temperature at probe ROIs (%)	
Bladder	35.5 (19.8, 50.7)
Rectum	42.0 (25.0, 55.6)
Vagina	37.9 (21.3, 56.0)
All probes	37.6 (21.8, 54.4)
HTV volume (cm^3^)	116.5 (74.9, 126.4)
Percentage of pixels used to calculate MR temperature in the HTV (%)	31.1 (18.6, 47.3)

* Net power corresponds to the radiated power.

### 3.1 MR temperature versus probe temperature

**Fig. 2:**
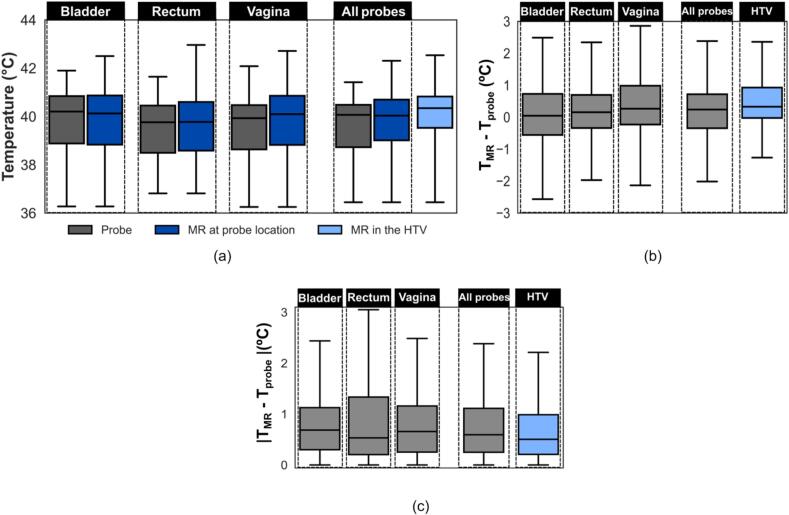
Comparison of probe and MR temperatures over the entire treatment duration, encompassing both the heating-up and steady-state phases. Data are presented separately for each probe location – bladder, rectum and vagina – as well as aggregated across all probe locations (“all probes”). (a) Comparison of median values for the entire patient cohort, showing temperatures measured by intraluminal probes (grey), MR thermometry at the probe locations (dark blue) and MR thermometry within the HTV (light blue). (b) Point-by-point comparison of median MR temperatures and median intraluminal probe temperatures at corresponding time points for each patient, , shown per probe location (grey) and for the HTV (light blue). (c) Comparison of median absolute errors between median MR temperatures and intraluminal probe temperatures, shown per probe location (grey) and for the HTV (grey). The temperature difference in the HTV is calculated relative to the median temperature measured by the probes across all locations for each time point. (MR: magnetic resonance; HTV: hyperthermia target volume; T_MR_: temperature measured with MR; T_probe_: temperature measured with intraluminal probes). (For interpretation of the references to colour in this figure legend, the reader is referred to the web version of this article.)

The comparison of probe and MR temperatures over the entire treatment, including both the heating-up and steady-state phases, is presented in Fig. 2. Median differences were 0 °C (bladder), 0.2 °C (rectum), 0.3 °C (vagina), 0.2 °C across all probes and 0.3 °C in the HTV (Fig. 2(b)). Median absolute errors were 0.7 °C (bladder), 0.5 °C (rectum), 0.7 °C (vagina), 0.6 °C across all probes, and 0.5 °C in the HTV (Fig. 2(c)). Steady-state results are shown in Fig. S4 (Supplementary Materials).

**Fig. 3:**
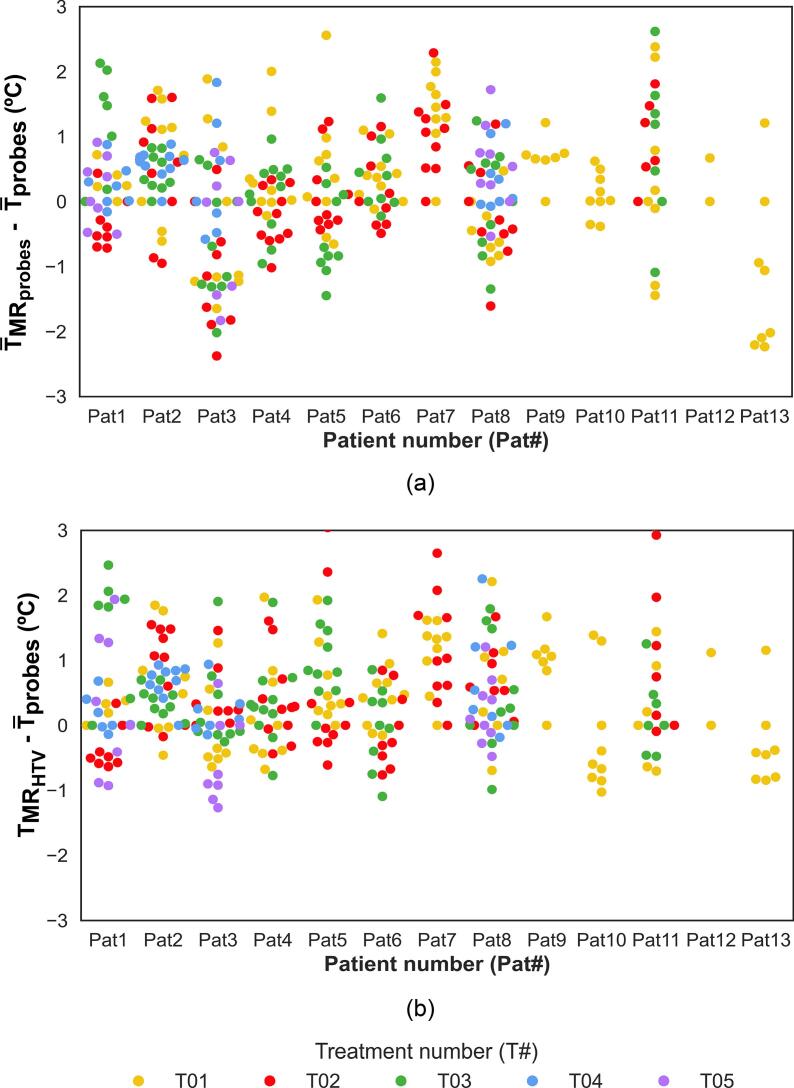
Point-by-point comparison of MR temperatures and the corresponding intraluminal probe temperatures at the same time points for each patient and treatment over the entire treatment. (a) Difference between mean MR temperatures at the probe locations and mean probe temperatures. (b) Difference between mean MR temperatures in the HTV and mean probe temperatures. (MR: magnetic resonance; HTV: hyperthermia target volume; $\overset{¯}{T}$_MR probes_: mean MR temperature over the probe locations; $\overset{¯}{T}$_probes_: mean intraluminal probe temperatures over the probe locations; $\overset{¯}{T}$_MR HTV_: mean MR temperature in the HTV).

The point-by-point comparison of MR and probe temperatures on a per-patient and per-treatment basis is presented in Fig. 3. Despite deviations, most values fall within clinically acceptable limits. Corresponding steady-state data are shown in Fig. S4 (Supplementary Materials).

### 3.2 Prediction role of MR temperatures in the HTV

The repeated measures correlations between probe temperatures and MR temperatures in the HTV for all data are presented in Table 2. Positive and significant correlations were observed in all comparisons, indicating that MR temperatures in the HTV resemble probe temperatures, particularly those measured at the vaginal probe.

**Table 2:** Repeated measures correlation between probe temperatures in the bladder, rectum and vagina and MR temperatures in the HTV. (MR: magnetic resonance; r_rm_: repeated measures correlation coefficient; CI: 95% confidence interval; HTV: hyperthermia target volume).

	Bladder probe temperature			Rectal probe temperature			Vaginal probe temperature		
	r_rm_	CI	*p*-value	r_rm_	CI	*p*-value	r_rm_	CI	*p*-value
MR temperature in the HTV	0.77	[0.72, 0.81]	<0.01	0.74	[0.69,0.79]	<0.01	0.79	[0.74, 0.82]	<0.01

**Fig. 4:**
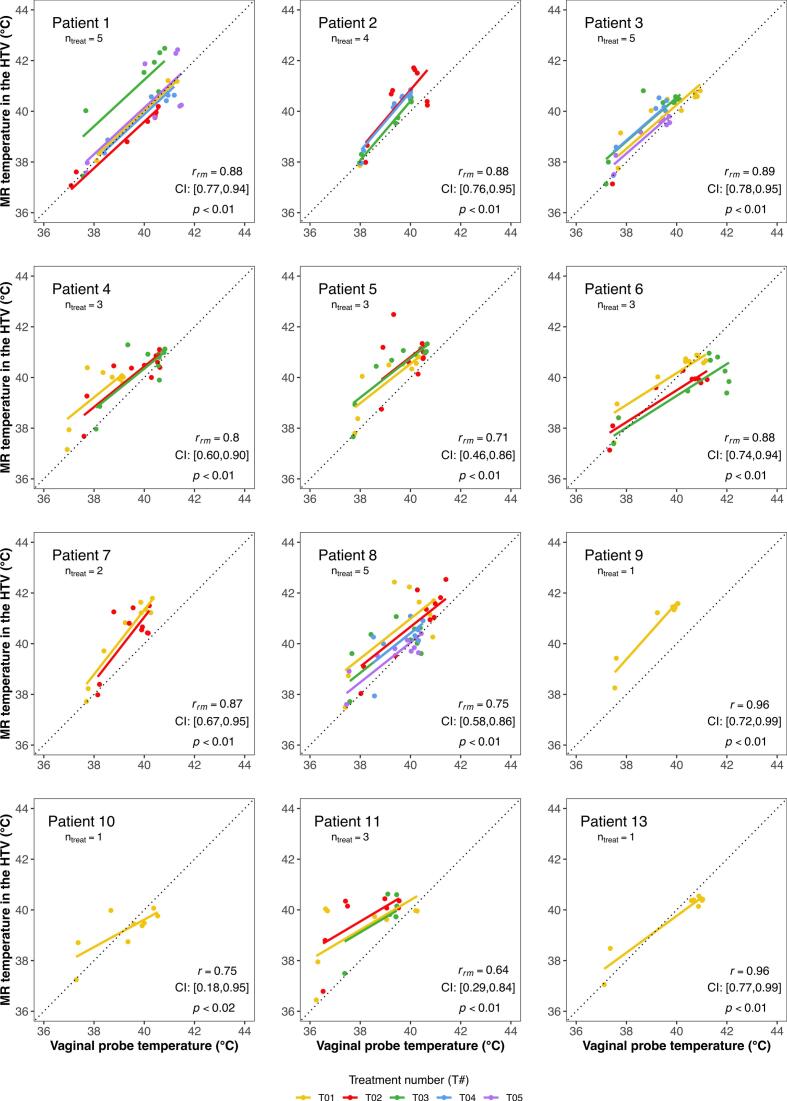
Repeated measures correlation between vaginal probe temperatures and MR temperatures in the HTV, for each patient for the entire treatment duration. The lines represent the fit for each hyperthermia treatment session per patient. For patients 9, 10 and 13, who completed only one MRgHT session, standard correlation is displayed. For the remaining patients, who underwent multiple MRgHT sessions, repeated measures correlation is shown. Each plot includes patient number, number of treatments, correlation coefficient (r_rm_ or r), 95 % confidence intervals (CI) and p-values. (MR: magnetic resonance; HTV: hyperthermia target volume; n_treat_: number of treatments; r_rm_: repeated measures correlation; r: correlation coefficient; CI: 95% confidence interval; p: p-value).

The repeated measures correlations between vaginal probe temperatures and MR temperatures in the HTV, for all treatments in the MR-compatible applicator per individual patient, are presented in Fig. 4. Equivalent bladder and rectal probe data are provided in the Supplementary Materials (Figs. S6 and S7). Patient 12 was excluded due to insufficient measurements (n = 2). For patients 9, 10 and 13, only one MRgHT treatment session was available, hence standard correlation analysis was applied. Significant positive correlations between MR and vaginal probe temperatures were observed across all patients. Notably, the slope of the correlation varied among patients, highlighting potential inter-patient differences.

Mixed-effects modelling revealed a slight negative effect of the number of pixels used in the calculation of MR temperatures in the HTV on MR temperatures within the HTV (β = −0.017, SE = 0.001, p < 0.01). Neither HTV volume nor subcutaneous fat volume demonstrated an association with MR temperature in the HTV (HTV volume: β = −1 × 10^−4^, SE = 9.7 × 10^−4^, p = 0.92; subcutaneous fat volume: β = 2 × 10^−5^, SE = 4.8 × 10^−5^, p = 0.65).

## 4 Discussion

This study comprehensively evaluated MR-based temperatures in LACC patients undergoing MRgHT. Firstly, good agreement was observed between MR thermometry and intraluminal probe temperatures at the same location, with a median absolute error within 0.7 °C (Fig. 2c). Although individual MR values do not always match probe readings (Figs. 3 and S5), most fall within the clinically accepted accuracy threshold [30], supporting its use for retrospective target temperature evaluation. However, if real-time treatment control is desired, further performance improvements are still required. All results were obtained by applying a threshold of 0 to 6 °C, which was acceptable for our dataset, as 43 °C was the maximum allowed temperature in healthy tissues (intraluminal locations). Subsequently, MR temperatures in the HTV, where probe measurements were unavailable, were further investigated. It was observed that MR temperatures in the HTV were within 0.5 °C of intraluminal temperatures for both group and point-by-point comparisons (Fig. 2 and Fig. S4). This difference is reassuring and supports the current practice using surrogate probe measurements for estimating temperatures within the HTV, when applying deep hyperthermia using the BSD2000-3D system [15]. Although not investigated it seems reasonable to extend the observation to other loco-regional deep hyperthermia systems.

Hyperthermia data is intrinsically hierarchical, i.e., several temperature measurements per treatment, several treatments and patients. Thus, we applied repeated measures correlation to avoid data loss by averaging across treatments and patients, which would have been necessary to avoid violating independence assumptions [35]. This is, to the best of our knowledge, the first time such analysis has been applied to hyperthermia temperature data. The obtained repeated measures correlations revealed a good linear relationship between probe and MR measurements in the HTV, with correlations between 0.74 and 0.79 (Table 2). This indicates that HTV temperatures measured with MR thermometry mirror those measured by the probes. However, it is important to recognise that this linear relationship largely reflects the general increase in temperature throughout the session, rather than exclusively demonstrating an intrinsic agreement between the two thermometry methods. Nonetheless, the primary aim of this analysis was to assess the quality and consistency of the relationship across different patients and sessions, prompting a patient-specific analysis. In this analysis, correlations between vaginal probe and MR temperatures in the HTV ranged from 0.64 to 0.96. These correlations were generally higher than those observed when the entire cohort data were analysed, which was expected given the lower variability of data points as all data originated from the same patient. Differences within the same patient, as observed for Patient 1 (T03) and Patient 6 (T01), were likely influenced by variations in mean net power, as these sessions involved an additional 50 W compared to the other sessions. Interestingly, the different estimations of correlation slopes in each patient are noteworthy. Patient 7, for instance, seems to be an “easy-to-heat” patient, compared to Patient 6. Perfusion and other factors may influence these variations and new studies should further investigate these and other patient-specific characteristics. Nevertheless, some of these findings were briefly introduced by Gellerman et al [27], who categorised easy-to-heat patients as those in which all tissues, including muscles, are heated well. In the same study, a correlation of 0.67 between probe temperatures and mean MR temperatures in the tumour volumes was reported, which is lower than the correlations found in this study. Other studies [22,23] have reported higher correlations (0.74–0.96), yet these come from a limited number of sarcoma patients (range 1–3). In contrast, our cohort is concerned with a different tumour entity and a higher number of patients and treatments, supporting the findings presented here.

Patient-specific characteristics, such as body size and tumour volume have been reported to influence thermal parameters, during hyperthermia [[37], [38], [39]]. Therefore, we assessed whether HTV or subcutaneous fat volume influence MR temperatures in the HTV. Neither showed a significant association, likely due to the limited patient number and variation in fat and tumour volumes. In contrast, the number of pixels used to compute MR temperatures in the HTV was found to negatively influence MR temperatures achieved in the HTV. However, this influence was small (β = −0.017).

The findings presented here should be interpreted in light of several limitations. Firstly, all published MR thermometry data, including ours, originate from a single hyperthermia system, the BSD2000-3D-MR, as only one manufacturer presently provides MRgHT capabilities. Consequently, both device-specific heating characteristics and patient selection – as only patients sufficiently slim to fit within the applicator were eligible for treatment – may have influenced the results. Caution is therefore advised when generalising these findings to other hyperthermia systems and broader patient populations. Secondly, MR temperatures were limited to the temperature range recorded by the intraluminal probes. However, certain regions within the HTV, such as necrotic areas, may have reached higher temperatures that were not captured due to the applied threshold. Increasing the upper threshold led to a greater increase in HTV temperature, while maintaining MR accuracy within 0.7 °C (Supplementary Materials, Fig. S8). Since no probes are placed within the target volume in current clinical practice, our approach of calibrating MR thermometry using probe data remains justified. Thirdly, MR temperature calculations relied on approximately 35 % of the total pixels within the probe and HTV regions of interest, a relatively low proportion. This reduction was due to gastrointestinal air artefacts which can introduce erroneous temperatures at tissue interfaces. Although some corrupted data points persisted despite the filtering process, leading to mismatches between MR and probe temperatures at specific time points, the overall agreement remained clinically acceptable. Several post-processing methods have been proposed to reduce such effects [[40], [41], [42]], but further improvements in accuracy and efficiency are required for routine clinical use.

Concluding, MR thermometry provides temperature estimates within HTV that show good agreement to intraluminal probe measurements. While this supports its feasibility for retrospective evaluation of treatment temperatures, its current performance is not yet sufficient for real-time treatment guidance and further improvements are required before clinical implementation.

## Funding statement

This research has received support from the European Union’s Horizon 2020 research and innovation program under the Marie Skłodowska-Curie (MSCA-ITN) grant “Hyperboost” project, no. 955625.

## Data availability statement

Research data are stored in an institutional repository and will be shared upon request to the corresponding author on request.

## Declaration of competing interest

The authors declare the following financial interests/personal relationships which may be considered as potential competing interests:

Gerard C. van Rhoon:

• Past President of the European Society for Hyperthermic Oncology, retired 2022
• Cofounder and shareholder Sensius BV
• Holds/submitted several patents on hyperthermia related technology
• Member executive committee Editorial Board Int. J. of Hyperthermia

Royalties or licenses

• E. Majorana Foundation; European School of Antennas; Various EU-Cost Actions
• Dr. Sennewald Medizintechnik Gmbh
• TU Munich
• Japanese STM
• SEOR
• IT’IS Foundation

Consulting fees

• WO 2013/028064 Al
• WO2020130824A1
• WO2022235155A1

Support for attending meetings and/or travel

• Received financial support to attend conferences from various companies, societies and charity
