# Supplementary material for: Magnetic resonance thermometry in the target volume versus intraluminal probe thermometry for hyperthermia treatment monitoring
Source: Phys Imaging Radiat Oncol. 2025 Jul 24;35:100812. doi: 10.1016/j.phro.2025.100812 (PMC12319249; doi:10.1016/j.phro.2025.100812)
Supplement: Supplementary Data 1 [file mmc1.docx]

**Supplementary materials**

**1. Patient characteristics**

Patient and tumour characteristics are shown in Table S1.

**Table S1:** Patient and tumour characteristics of the LACC patients included in this study. Data are presented as frequency count, mean ± standard deviation (mean ± std) for age and median (iqr) for subcutaneous fat volume due to non-normal distribution.

| **Characteristic** | **Value** |
| --- | --- |
| Age (years) | 52.2 ± 15.6 |
| Histology  Adenocarcinoma  Squamous cell carcinoma | 3  10 |
| FIGO stage*  I  II  III  IVA | 2  5  4  2 |
| Subcutaneous fat volume (cm^3^) | 4591 (3764, 5712) |

*FIGO stage 2008

**2. MRgHT treatment sessions**

The reasons for using non-MR applicator rather than the MR-compatible applicator for all treatments of all patients are summarised in Figure S1. In brief, although the MR-compatible applicator was preferred to enable MR thermometry, its use was limited by patient preference (n_treat_ = 21) and occasionally unavailability due to technical problems (n_treat_ = 6). Consequently, some patients received the remaining treatments using the BSD-2000-3D non-MR system. Importantly, no patient was switched to the BSD-2000 non-MR system.

**Figure S1:** Flowchart of patient and treatment inclusion for the study. A total of 14 patients undergoing hyperthermia with at least one MRgHT treatment were initially considered. One patient was excluded due to a tumour type other than LACC. The total 13 included patients underwent 64 hyperthermia treatments of which only 37 were MRgHT treatments. The reasons for replacing the MR treatment with non-MR treatment are listed. The same applicator, the BSD-2000-3D, was used for both MR and non-MR treatments.

**3. Delineations and image registration**

The baseline MR image from each treatment session was used to locate the catheters containing the thermal probes Due to the difficulty in precisely identifying the probes in MR images, circular regions of interest (ROIs) were drawn around each probe location in every slice. These ROIs had a diameter of 1.37 cm, corresponding to an area of 1.47 cm^2^ [30].

The hyperthermia target volume (HTV) is defined as the clinical target volume (CTV) as delineated by the radiation oncologist and includes the cervix, uterus, parametria and upper 2 cm of the vagina. In case of a FIGO IIIA, the entire vagina was included in the volume. The HTV is delineated in the computed tomography (CT) images used for hyperthermia treatment planning.

To transfer the HTV from the planning CT to the magnetic resonance (MR) image, a rigid registration approach using MIM software (version 7.1.6, MIM Software Inc., Cleveland, OH, USA) was implemented. Figure S2 shows the procedure followed. Bone structures were visible and consistently positioned across both image modalities and therefore served as primary reference landmarks to ensure robust initial alignment of the datasets. Following the bone-based alignment, the registration was further refined by incorporating tumour markers visible on both imaging modalities. These markers were crucial for improving alignment accuracy in soft-tissue regions, particularly around the HTV. The final registration was peer-reviewed to ensure consistency and adjustments were made if necessary (Figure S2). Through this process, the HTV delineation, originally performed on the CT images, was reliably transferred to the MR dataset.


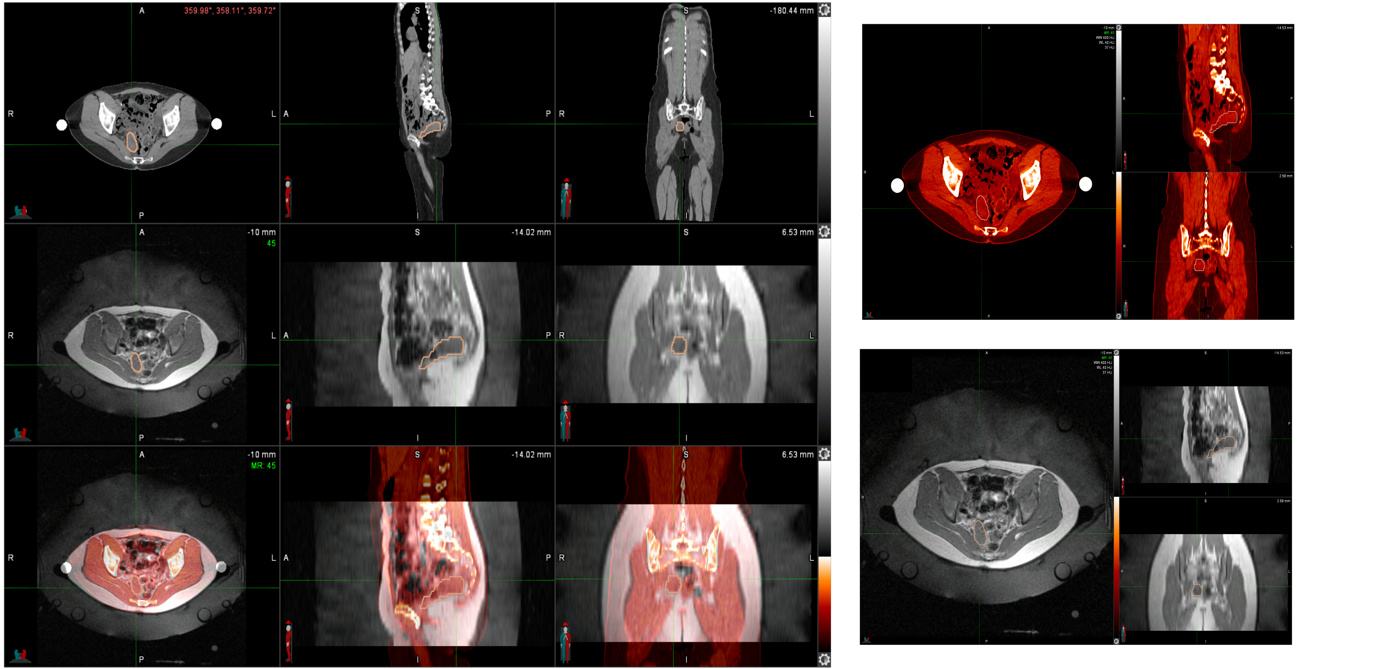


**Figure S2:** Rigid registration procedure. Left panel shows the alignment between the CT (first row) and MR images (second row), with the overlaid position of the two datasets displayed in the third row. Necessary adjustments were made to ensure proper alignment of bone structures. The final aligned images were reviewed individually (right panel, with CT on top and MR below) to identify tumour markers, followed by any required adjustments. HTV is delineated in light pink.

**4. Intraluminal and MR thermometry**

We analysed the intraluminal probe temperatures by calculating the mean temperature along the entire length of each probe track. To ensure accurate comparison, we identified the anatomical regions corresponding to these probe tracks within the MR images. MR temperatures were then extracted from these specific locations on the images, ensuring that they matched the anatomical positions of the probes (cf. Delineations and image registration). The average temperatures from the probes were subsequently compared to the corresponding averaged MR temperatures at the same anatomical locations for the same time points during treatment.

**4.1. MR thermometry processing**

Various methods have been proposed for MR thermometry processing, being the proton resonance shift method (PRFS) the most adopted method, as the proton resonance varies linearly over a large temperature range and is independent of tissue type (with the exception of fat) [1,2]. Therefore, with the PRFS relative temperature changes with regard to a reference image can be detected. By subtracting a reference phase image from a phase image at the time point of interest, the change in phase (phase shift) can be determined and this is proportional to the temperature change, as follows:

$$\Delta T=\frac{\Delta\varphi}{\gamma\alpha B_{0}TE} = \frac{\varphi_{n}-\varphi_{ref}}{\gamma\alpha B_{0}TE}$$

where ﻿$\Delta\varphi$ corresponds to the phase shift given by $\varphi_{n}-\varphi_{ref}$. $\varphi_{n}$ corresponds to the phase image at the time of interest and $\varphi_{ref}$ corresponds to the reference phase image, taken at baseline conditions, i.e., right before starting the heating. $\gamma$is the gyromagnetic ratio (﻿267.5 × 10^6^ rad/T·s); $\alpha$ is the change coefficient for PRFS (﻿−0.001 ppm/°C); B_0_ is the magnetic field strength of the MR scanner ﻿(1.5 T) and ﻿TE is the echo time equal to 19.1 ms.

Commonly, errors in temperature predictions can occur due to phase wrapping - phase difference between successive MR images exceeds 2𝜋 radians - and B_0_-drift [3]. Phase wrapping was corrected by applying a phase-unwrapping algorithm, previously described by Dymerska et al. [4]. B_0_-drift was corrected by using fat-like tube references placed in the MR-compatible hyperthermia system. Additionally, it has been shown that combining the fat-tube references with the patient’s subcutaneous fat improves the correction and consequently measurement accuracy [5,6]. Thus, we used the combination of the two types of reference points for the drift correction: (a) four fat tubes placed on the edges of the hyperthermia system and (b) subcutaneous fat from each patient (Figure 1). The correction consisted of a two-dimensional (2D) polynomial fitting through both reference types that was applied to the whole field of view [7,8]. Finally, various thresholds, relative to the baseline temperature, were tested to identify the one that most effectively removed unrealistic data caused by confounders. This was validated by comparing MR temperatures at the probe locations with the actual probe temperatures (Figure S3(a)). Moreover, point-by-point temperature differences were calculated to determine the MR thermometry error in regions where probes were present ($T_{{MR}_{i}} - T_{{probe}_{i}} for i = bladder, rectum and vagina$). For the HTV, the temperature difference was calculated by subtracting the average probe temperature across all locations. These results are presented in Figure S3(b). Based on these data, a threshold of [0, 6] ºC was selected for our dataset, as temperatures exceeding 43 ºC were not recorded by the intraluminal probes. Additionally, this threshold resulted in the lowest median MR thermometry error across all probe locations (≤ 0.2 ºC).


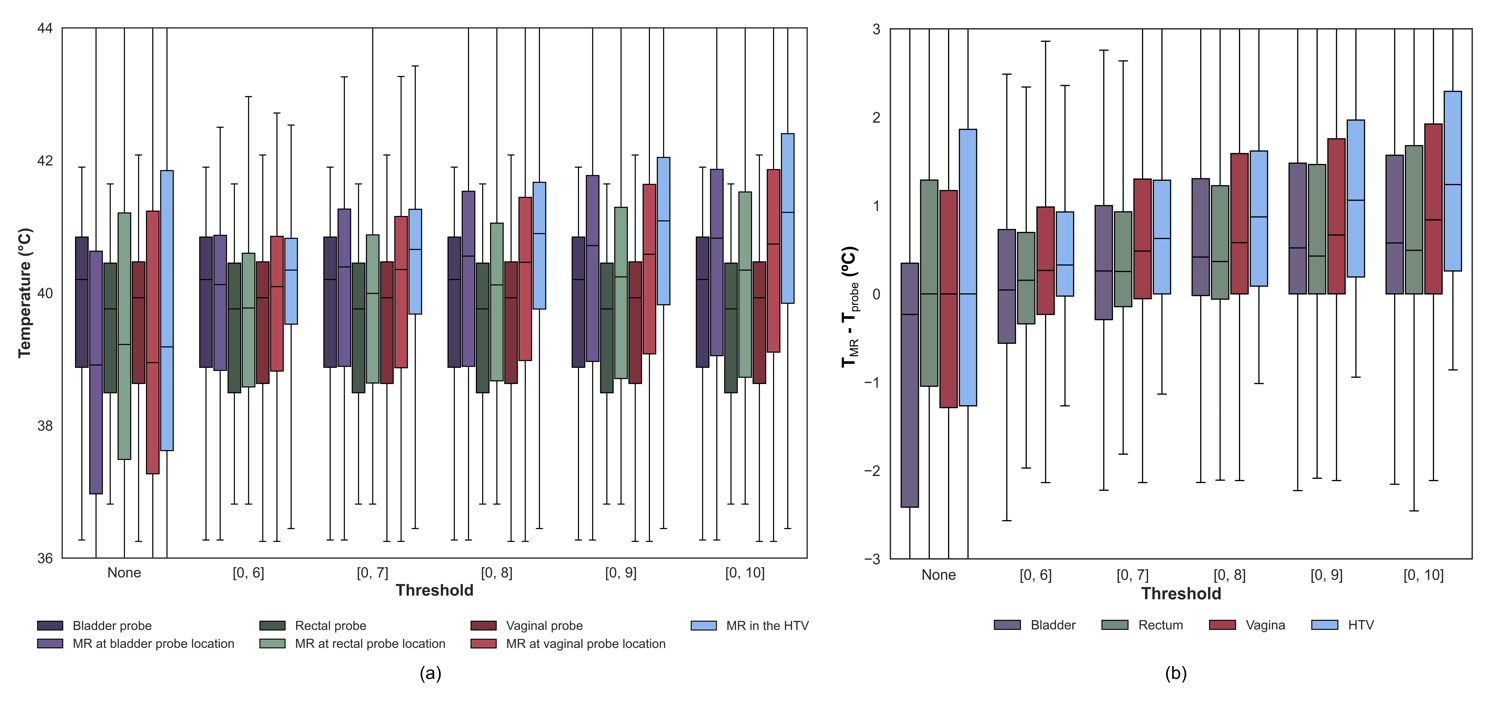


**Figure S3**: Comparison of probe and MR temperatures over the entire treatment duration, encompassing both the heating-up and steady-state phases. Data are presented separately for each probe location – bladder, rectum and vagina. (a) Comparison of median values for the entire patient cohort, showing temperatures measured by intraluminal probes (dark colours), MR thermometry at probe locations (light colours) and MR thermometry in the HTV (light blue), shown for various filtering thresholds. (b) Point-by-point comparison of median MR temperatures and the corresponding median intraluminal probe measurements at the same time points for each patient. The temperature difference in the HTV is calculated relative to the median temperature measured by the probes across all locations for each time point. (MR: magnetic resonance; HTV: hyperthermia target volume; T_MR_: temperature measured with MR; T_probe_: temperature measured with intraluminal probes).

**5. Results**

**Table S2:** Temperature-related parameters for both probe and MR thermometry. Data are presented as frequency count, mean ± standard deviation (SD) or median (interquartile range, IQR) as appropriate. Hyperthermia characteristics were obtained for the entire treatment duration, except when steady-state is mentioned. Values for steady-state correspond to the values during the therapeutic phase of the treatment, i.e., the last 60 minutes of the hyperthermia treatment. (MR: magnetic resonance; HTV: hyperthermia target volume).

| **Parameter** | **Value** **(mean ± std or**  **median (IQR))** |
| --- | --- |
| Median intraluminal probe temperature for steady-state (ºC)  bladder  rectum  vagina  all probes | 40.7 (40.3 - 41.1)  40.3 (39.9 - 40.8)  40.3 (40.0 - 40.7)  40.5 (40.0 - 40.9) |
| Median MR temperature at probe location for steady-state (ºC)  bladder  rectum  vagina  all probes | 40.5 (39.2 - 41.1)  40.1 (39.5 - 40.8)  40.4 (39.7 - 41.2)  40.3 (39.6 - 41.0) |
| Median MR temperature in the HTV for steady-state (ºC) | 40.6 (40.2 - 41.1) |

The comparison of probe and MR temperatures over the steady-state phases is presented in Figure S4. The median differences between MR and probe temperatures were -0.3 °C, 0 °C, 0.1 °C, for the bladder, rectum and vagina, respectively. Across all probe locations, the median difference was 0 °C, while the MR temperature within the HTV was within 0.2 °C from the probe temperature across all locations (Figure S4(b)). Median absolute errors were 0.8 °C, 0.7 °C, 0.8 °C for the bladder, rectum vagina and across all probe locations, respectively, while within the HTV was 0.5 °C (Figure S4(c)).

**
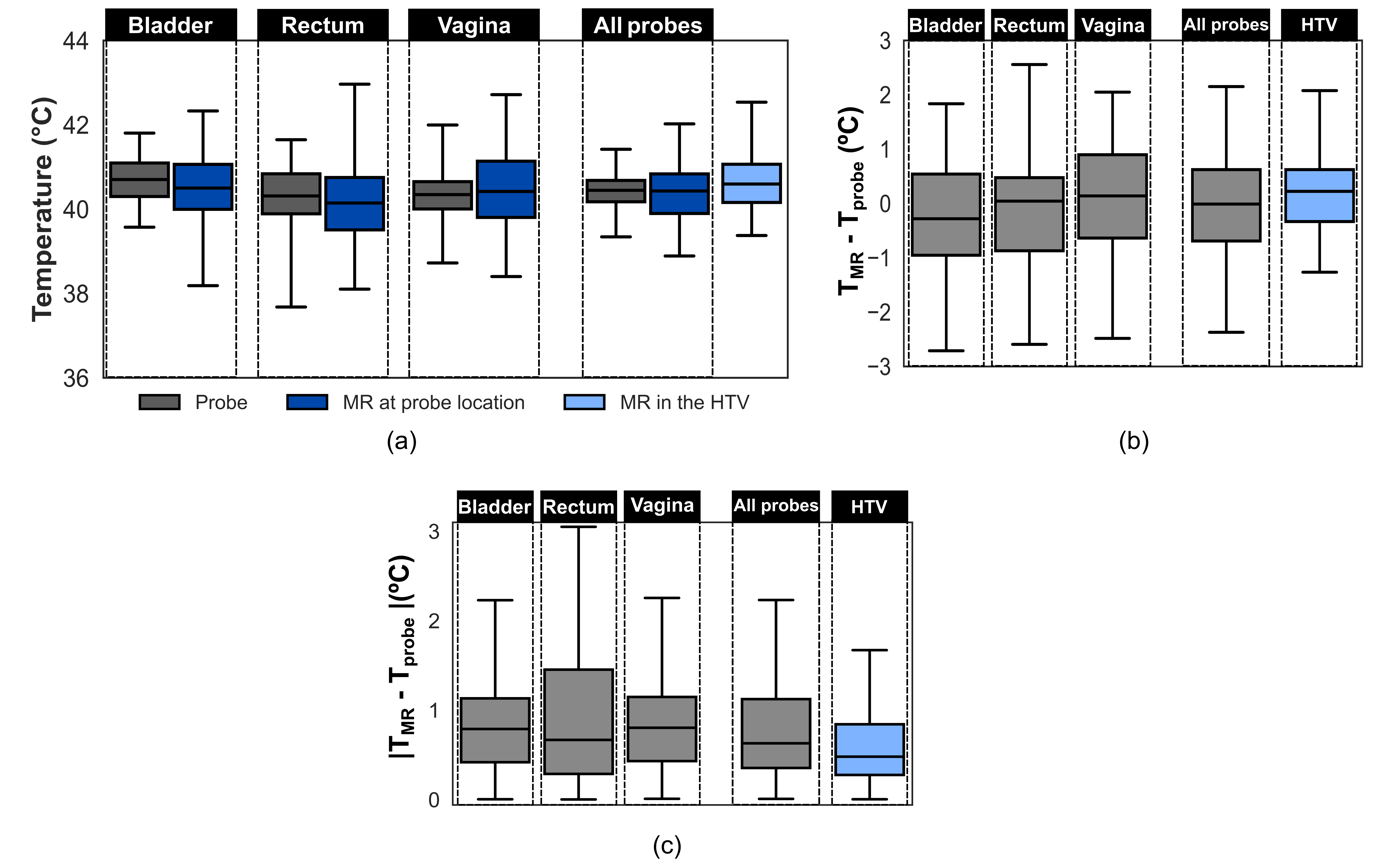
Figure S4:** Comparison of probe and MR temperatures over steady-state phase. Data are presented separately for each probe location – bladder, rectum and vagina – as well as aggregated across all probe locations ("all probes"). (a) Comparison of median values for the entire patient cohort, showing temperatures measured by intraluminal probes (grey), MR thermometry at the probe locations (dark blue) and MR thermometry within the HTV (light blue). (b) .Point-by-point comparison of median MR temperatures and median intraluminal probe temperatures at corresponding time points for each patient, , shown per probe location (grey) and for the HTV (light blue). (c) Comparison of median absolute errors between median MR temperatures and intraluminal probe temperatures, shown per probe location (grey) and for the HTV (grey). The temperature difference in the HTV is calculated relative to the median temperature measured by the probes across all locations for each time point. (MR: magnetic resonance; HTV: hyperthermia target volume; T_MR_: temperature measured with MR; T_probe_: temperature measured with intraluminal probes).

Figure S5 presents the point-by-point comparison of MR and probe temperatures during the steady-state phase, on a per-patient, per-treatment basis. While variation remains present, the majority of measurements lie within a clinically acceptable range. As with the full-duration data, agreement is stronger in the HTV - reflecting the primary aim of this analysis, which was to assess the feasibility of MR thermometry in regions lacking direct temperature measurements.

**
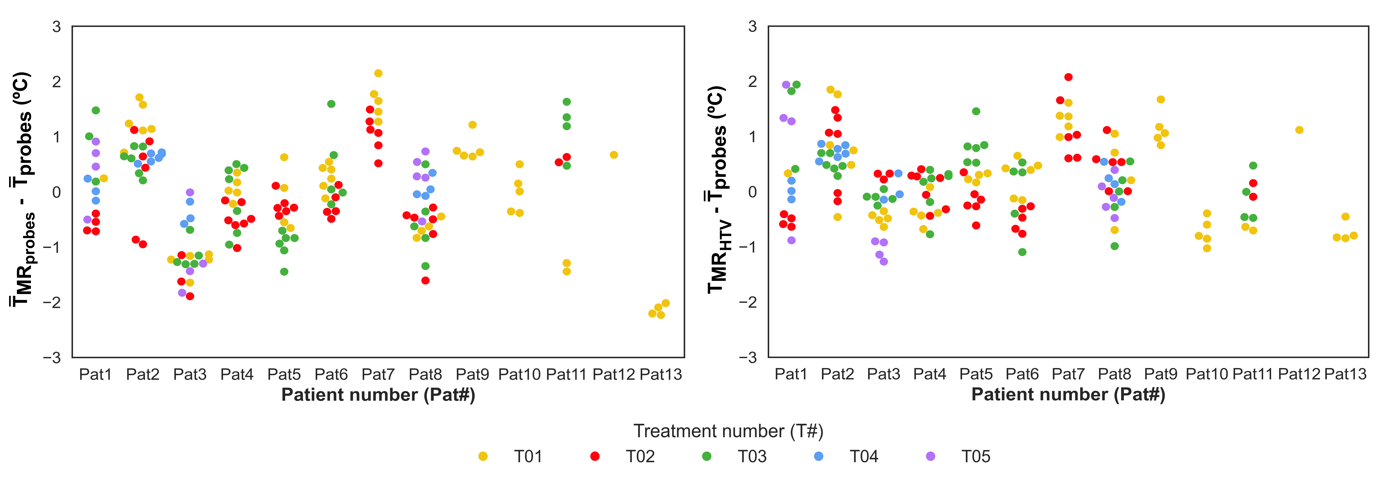
Figure S5:** Point-by-point comparison of MR temperatures and the corresponding intraluminal probe temperatures at the same time points for each patient and treatment over the steady-state phase. (a) Difference between mean MR temperatures at the probe locations and mean probe temperatures. (b) Difference between mean MR temperatures in the HTV and mean probe temperatures. (MR: magnetic resonance; HTV: hyperthermia target volume; $\bar{T}$_MR probes_: mean MR temperature over the probe locations; $\bar{T}$_probes_: mean intraluminal probe temperatures over the probe locations; $\bar{T}$_MR HTV_: mean MR temperature in the HTV).

Repeated measures correlation results between bladder probe temperatures and MR temperatures in the HTV are shown Figure S6. As seen for the vaginal probe, correlation values for bladder were also good, ranging between 0.57 and 0.95, indicating a good linear relation between probe temperatures and MR temperatures in the HTV.

**
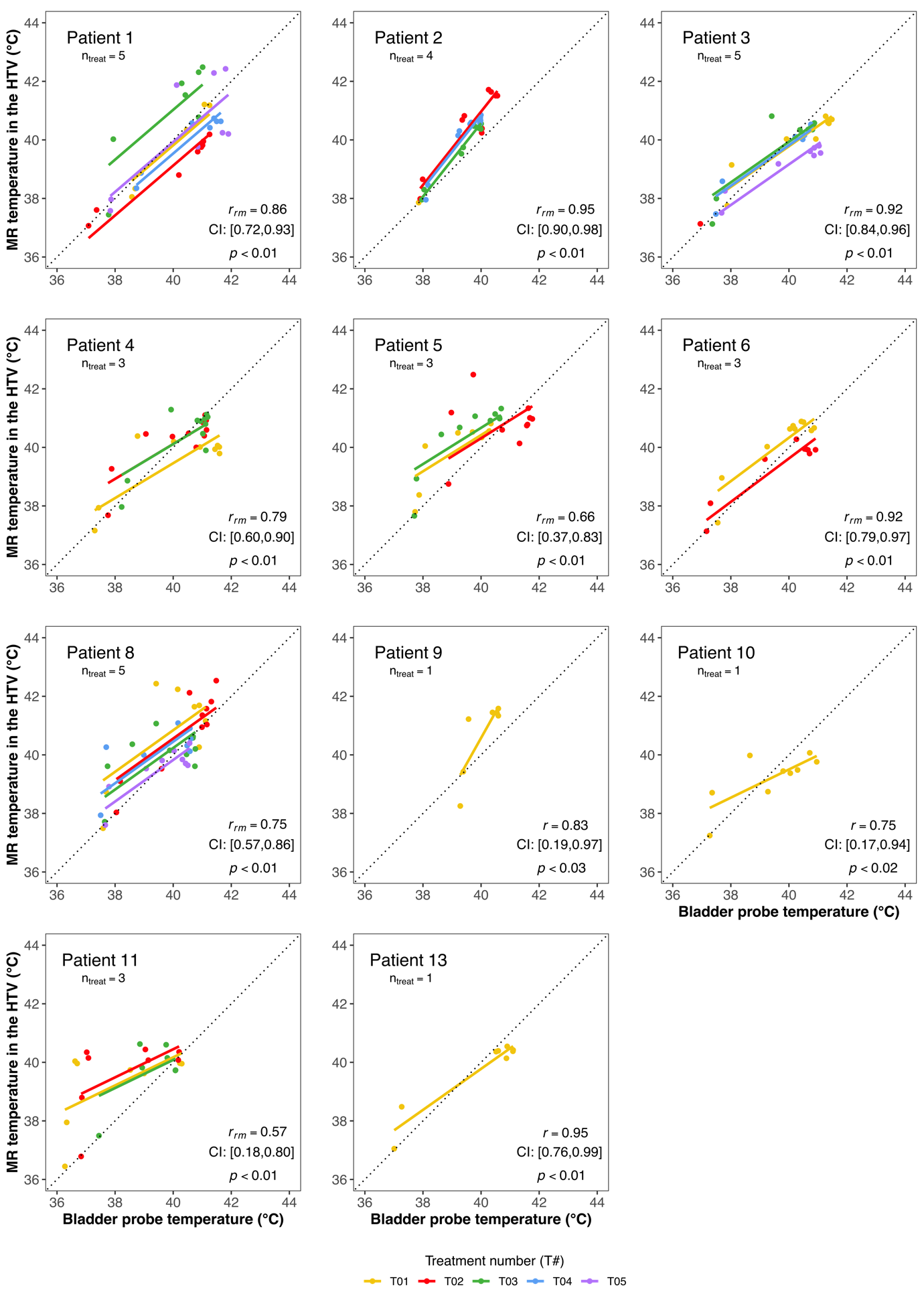
Figure S6:** Repeated measures correlation between bladder probe temperatures and MR temperatures in the HTV, for each patient. The lines represent the fit for each hyperthermia treatment session per patient. Patient 7 is not shown since no bladder probe measurements were performed. For patients 9, 10 and 13, who completed only one MRgHT session, standard correlation is displayed. For the remaining patients, who underwent multiple MRgHT sessions, repeated measures correlation is shown. Each plot includes patient number, number of treatments, correlation coefficient (r_rm_ or r), 95% confidence intervals (CI) and p-values. (MR: magnetic resonance; HTV: hyperthermia target volume; ntreat: number of treatments; rrm: repeated measures correlation; r: correlation coefficient; CI: 95% confidence interval; p: p-value).

Repeated measures correlation results between rectal probe temperatures and MR temperatures in the HTV are shown Figure S7. Correlation values for bladder were good, ranging between 0.59 and 0.96, indicating a good linear relation between probe temperatures and MR temperatures in the HTV.


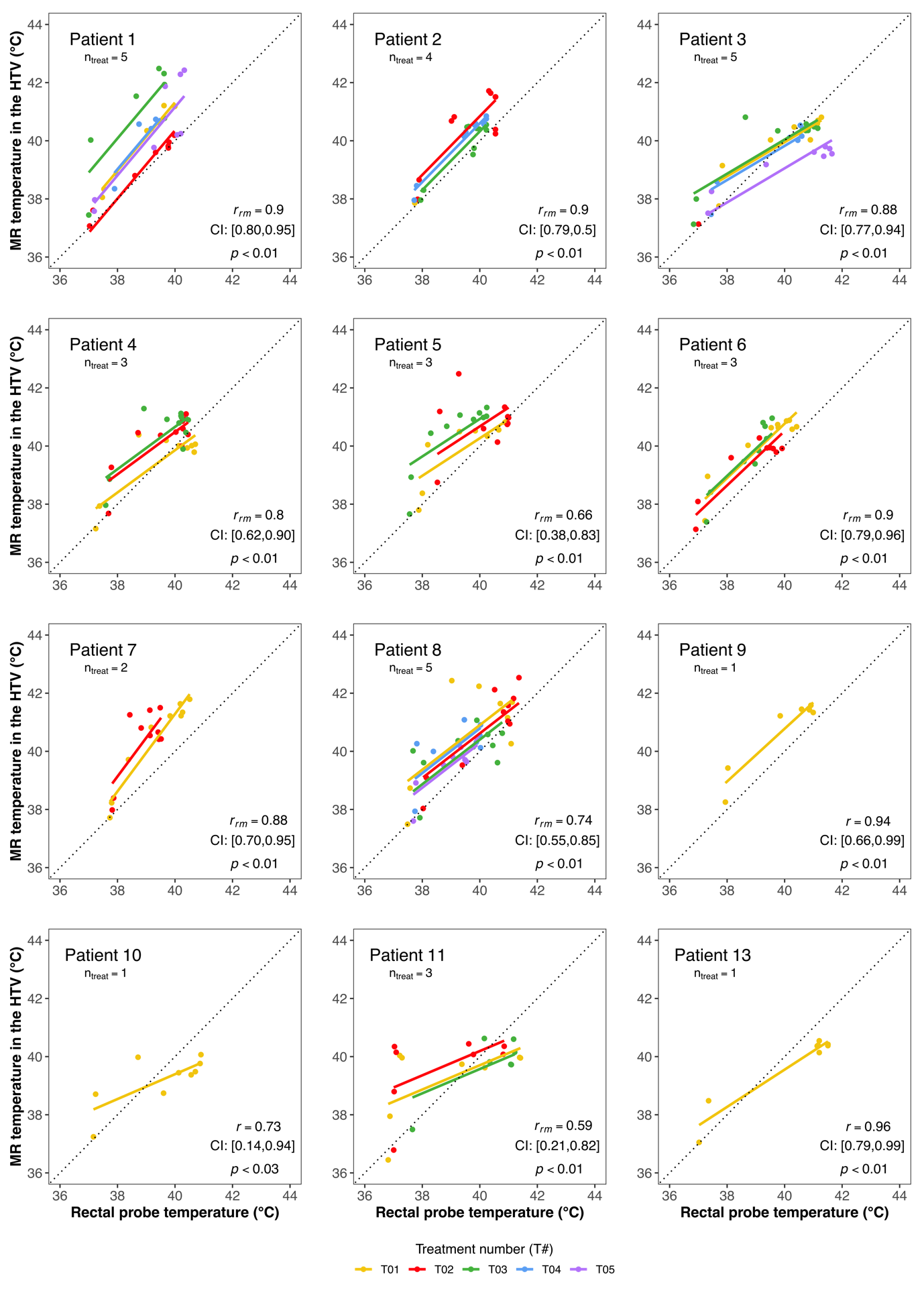


**Figure S7:** Repeated measures correlation between rectal probe temperatures and MR temperatures in the HTV, for each patient. The lines represent the fit for each hyperthermia treatment session per patient. For patients 9, 10 and 13, who completed only one MRgHT session, standard correlation is displayed. For the remaining patients, who underwent multiple MRgHT sessions, repeated measures correlation is shown. Each plot includes patient number, number of treatments, correlation coefficient (r_rm_ or r), 95% confidence intervals (CI) and p-values. (MR: magnetic resonance; HTV: hyperthermia target volume; n_treat_: number of treatments; r_rm_: repeated measures correlation; r: correlation coefficient; CI: 95% confidence interval; p: p-value).

Figure S8 illustrates the variation in temperature differences (point-by-point comparison) across all probe locations and within the HTV as a function of the tested thresholds. The results indicate that increasing the threshold leads to a more pronounced rise in HTV temperature compared to the corresponding increase in MR thermometry error. This suggests that while higher thresholds allow for capturing elevated temperatures within the HTV, they do not substantially compromise the accuracy of MR thermometry.

**
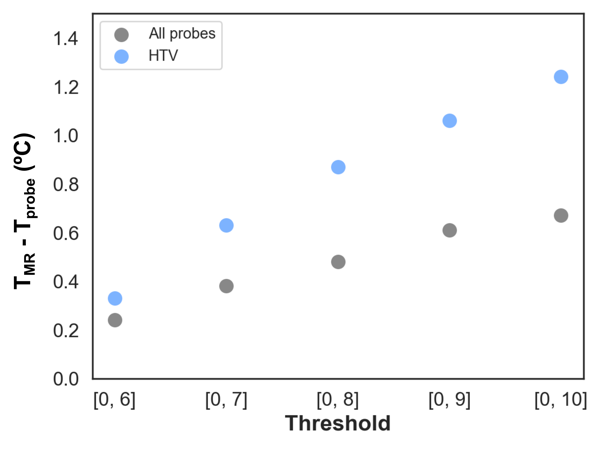
**

**Figure S8:** Variation in temperature differences (point-by-point comparison) across all probe locations and within the HTV as a function of the tested thresholds. Temperature difference in all probes calculated relative to the average MR and probe temperature across all locations (grey). Temperature difference in the HTV calculated relative to the average probe temperature across all locations. (MR: magnetic resonance; HTV: hyperthermia target volume; T_MR_: temperature measured with MR; T_probe_: temperature measured with intraluminal probes).

**References**

1. Wlodarczyk W, Hentschel M, Wust P, Noeske R, Hosten N, Rinneberg H, et al. Comparison of four magnetic resonance methods for mapping small temperature changes. *Phys. Med. Biol.* 1999; *44*, 607–624. https://doi.org/10.1088/0031-9155/44/2/022.

2. Ishihara Y, Calderon A, Watanabe H, Okamoto K, Suzuki Y, Kuroda K, et al. A precise and fast temperature mapping using water proton chemical shift. *Magn. Reson. Med.* 1995; *34*, 814–823. https://doi.org/10.1002/mrm.1910340606.

3. El-Sharkawy AEM, Schär M, Bottomley PA, Atalar E. Monitoring and correcting spatio-temporal variations of the MR scanner’s static magnetic field. *Magn. Reson. Mater. Physics, Biol. Med.* 2006; *19*, 223-236. https://doi.org/10.1007/s10334-006-0050-2.

4. Dymerska B, Eckstein K, Bachrata B, Siow B, Trattnig S, Shmueli K, et al. Phase unwrapping with a rapid opensource minimum spanning tree algorithm (ROMEO). *Magn. Reson. Med.* 2021; *85*, 2294-2308. https://doi.org/10.1002/mrm.28563.

5. Vilasboas-Ribeiro I, Curto S, van Rhoon GC, Franckena M, Paulides MM. MR thermometry accuracy and prospective imaging-based patient selection in MR-guided hyperthermia treatment for locally advanced cervical cancer. *Cancers* 2021; *13*, 3503. https://doi.org/10.3390/cancers13143503.

6. Karkavitsas SN, Göger-Neff M, Kawula M, Sumser K, Zilles B, Wadepohl M, et al. Evaluation of magnetic resonance thermometry performance during MR-guided hyperthermia treatment of soft-tissue sarcomas in the lower extremities and pelvis. *Int. J. Hyperth.* 2024; *41*, 2405105. https://doi.org/10.1080/02656736.2024.2405105.

7. Rieke V, Pauly KB. MR thermometry. *J. Magn. Reson. Imaging* 2008; *27*, 376–390. https://doi.org/10.1002/jmri.21265.

8. Wu M, Mulder HT, Baron P, Coello E, Menzel MI, van Rhoon GC, et al. Correction of motion-induced susceptibility artifacts and B0 drift during proton resonance frequency shift-based MR thermometry in the pelvis with background field removal methods. *Magn. Reson. Med.* 2020; *84*, 2495-2511. https://doi.org/10.1002/mrm.28302.
